# Recent advances in benign gynecological laparoscopic surgery

**DOI:** 10.12703/r/10-60

**Published:** 2021-07-26

**Authors:** Lior Levy, Jim Tsaltas

**Affiliations:** 1 Gynaecological Endoscopy and Endometriosis Surgery, Department of Obstetrics and Gynaecology Monash Health, Monash University, Melbourne, Victoria, Australia

**Keywords:** Endometriosis, Laparoscopic entry techniques, Single incision Laparoscopic Surgery, Enhanced Recovery After Surgery

## Abstract

Minimally invasive surgery continues to transform the field of gynecological surgery and is now the standard of care for the surgical treatment of many diseases in gynecology. Owing to minimally invasive surgery’s clear advantages, new advances in technology are being employed rapidly and enabling even the most complicated procedures to be performed less invasively. We examine recent literature on minimally invasive surgical innovations, advances, and common practices in benign gynecology that, from our point of view, made an impact on the way laparoscopic surgery is performed and managed in the last decade.

## Introduction

The introduction of endoscopy into surgical practice is one of the biggest success stories in the history of medicine. The first description of an endeavor to reflect light into the human body in order to become conversant with its innermost structure is found in the Babylonian Talmud (about 200 BCE; Niddah, V.65.b). In that tract, there is a description of a lead pipe (*siphopherot*) with an inwardly tilted mouthpiece furnished with a *mechul* (wooden drain). Both devices were introduced into the vagina to determine whether bleeding originated in the uterus or the vagina^1^.

The credit for the first true laparoscopy on a human goes to Hans Christian Jacobaeus from Stockholm, who coined the term “laparoscopy” and in 1910 described his technique for the inspection of the human peritoneal, thoracic, and pericardial cavities^2^. Only four decades later, gynecological laparoscopy was introduced, developed, and used routinely by European pioneers, such as Raoul Palmer and Hans Frangenheim^2^. Patrick Steptoe, considered one of the pioneers of *in vitro* fertilization, learned the surgical technique from Palmer and Frangenheim and in 1967 published the first English-language textbook describing laparoscopic surgical techniques, which contributed the widespread dissemination of this technique to the English-speaking world^3^.

Initially, laparoscopy was used for diagnosis and simple therapeutic procedures such as tubal ligation and fenestration of benign ovarian cysts but gradually became more sophisticated owing largely to the pioneering work of Kurt Semm, who developed numerous laparoscopic instruments that contributed to the development of laparoscopic surgery as we know it today^4^.

Developments in the techniques of operative laparoscopy and operative hysteroscopy have had a major impact on the specialty of gynecological surgery. At present, minimally invasive surgery is the standard of care for the treatment of many gynecological conditions and the most frequently performed gynecological surgical approach in Western countries.

The proven benefits of minimally invasive surgery, such as decreased blood loss, decreased postoperative pain, decreased perioperative complications, shorter hospitalization, and faster recovery when compared with laparotomy^5,6^, are driving the rapid introduction and dissemination of novel technologies and the increasing ability to perform even the most complex procedures less invasively. However, as new technologies are disseminated, it is imperative that these techniques be critically evaluated to ensure that perioperative morbidity and outcomes are at least equivalent to those of traditional surgical approaches.

In this article, we review the current literature on minimally invasive surgical innovations, advances, and common practices in benign gynecology that, from our point of view, made an impact on the way laparoscopic surgery is performed and managed in the last decade.

Considering the vast number of new innovations in laparoscopic surgery, we decided to include key topics that have impacted laparoscopic surgery at all levels:

- *Preoperative evaluation in laparoscopy*: the role of ultrasound and magnetic resonance imaging (MRI) in the assessment of endometriosis and related pelvic conditions
- *Advances in devices and techniques*: laparoscopic entry techniques, morcellation in laparoscopic surgery, and barbed sutures in laparoscopic surgery
- *Advances in surgical approaches*: single-incision laparoscopic surgery (SILS) and transvaginal natural orifice transluminal endoscopic surgery (vNOTES)
- *Advances in postoperative management and reduction of complications*: enhanced recovery after surgery (ERAS) in minimally invasive gynecological surgeries
- *Education in laparoscopic surgery*: the role of simulation in laparoscopic surgery training
- Finally, we have included a section on the *challenges and limitations in laparoscopic surgery*.

Importantly, we decided to exclude innovations in robot-assisted surgery from this review since we believe that this topic is extremely broad and deserves a separate discussion.

## Selection criteria and methods

We selected the topics to be discussed after consultation with the other surgeons in our department. We then conducted a literature search (Medline 2010–2021) separately for each section of the article. We preferred literature from the last five years whenever possible. Because of the broad nature of the topics covered, we generally preferred to cite good-quality reviews rather than the original articles.

## Preoperative imaging assessment in endometriosis and related pelvic conditions

The officially recommended gold standard in many guidelines for diagnosing endometriosis is laparoscopy with tissue biopsy^7,8^. Despite this, most gynecological societies advocate empiric medical treatment before tissue diagnosis^9,10 ^.

Recent advances in imaging technology, techniques, and protocols of both ultrasound and MRI assessments challenge the need for surgical diagnosis and aiding in preoperative planning^11^.

The potential advantages of highly detailed preoperative imaging assessment are clear: classic findings of endometriosis found upon imaging lead to diagnosis and the pursuit of non-surgical treatment, if suitable. If surgery is needed, understanding the location and extent of lesions identified by imaging allows more thorough planning prior to the procedure as well as better patient counselling. It also enables the assembly of a multidisciplinary team of surgeons, such as a gynecological surgeon specializing in the minimally invasive treatment of endometriosis and a colorectal surgeon in the case of endometriosis lesions involving the bowel. Improved imaging also allows the patient to make a properly informed decision and give appropriate consent.

### Advanced ultrasound imaging

Ultrasound is considered the first line of choice since it has many potential advantages over MRI: it has fewer contraindications, is less expensive and less time-consuming, and (owing to the dynamic aspects of ultrasound) is useful in the assessment of adhesions and site-specific tenderness^12,13^.

A Cochrane review and other studies have demonstrated high diagnostic accuracy for ultrasound in detecting endometriomas, deep infiltrative endometriosis (DIE), and pouch of Douglas obliteration. Specifically, the mean sensitivity and specificity for these three features are 93%, 79%, and 83% and 96%, 94%, and 97%, respectively^14–16^.

In 2016, the International Deep Endometriosis Analysis Group (IDEA) detailed their proposal for the systematic sonographic evaluation of suspected endometriosis^17^. The aim was to conduct an extensive examination of the pelvis by separating it into discrete sections and including an assessment of soft markers for deep endometriosis as well as mobility of the pelvis structures. Together with other suggested advanced endometriosis ultrasound systematic protocols based on the IDEA approach, such as the one proposed recently by Leonardi and Condous^18^, focused, advanced ultrasound studies by experienced operators are becoming the mainstay in the modern management of DIE and an important part of the preoperative evaluation^11^. A recent study concluded that an endometriosis-focused ultrasound may negate a two-step surgery pathway, including diagnostic surgery, and save money to the health-care system as well^19^.

### Newer modalities in ultrasound

A published systematic review and meta-analysis^20^ reported sensitivities of 94.6% and 99% and specificities of 97.1% and 99.3% using rectal water contrast for the diagnosis of DIE involving the rectosigmoid and rectovaginal space, respectively. This technique involves the instillation of saline or sterile water into the rectum through a catheter to create an acoustic window to visualize implants through a transvaginal sonographic approach. Three-dimensional (3D) ultrasound is a newer modality that has made major advances in the imaging evaluation of endometriosis and mainly adenomyosis with high accuracy^20^.

### Magnetic resonance imaging for endometriosis 

MRI is often used for preoperative staging of the disease with high accuracy as well and is considered a second-line imaging technique after ultrasound imaging in the evaluation of endometriosis because of higher costs and reduced availability worldwide^21^. The decision to use this modality should be based on the available expertise, in cases when it is not possible to perform transvaginal ultrasound, status of existence of pelvic organs (that is, previous hysterectomy or oophorectomy), or when diagnosis is not clear with ultrasound findings, and more information is needed (i.e better understanding of lesion characteristics and/or location). When compared in a recent meta-analysis by Guerriero *et al*., ultrasound and MRI showed similar performance when deep endometriosis in the rectosigmoid, uterosacral ligaments, and rectovaginal septum was assessed^12^.

Another recent study from a prospective observational cohort study, which involved the IDEA consensus for ultrasound and its modified version for MRI^22^, investigated the diagnostic accuracy of transvaginal ultrasound and MRI in the mapping of deep pelvic endometriosis. It showed that both imaging techniques had overall good agreement with the reference standard (intraoperative findings with histopathological confirmation) in the detection of deep pelvic endometriosis.

### Magnetic resonance imaging for the assessment of fibroids

Ultrasound is often the first choice of imaging modality for the detection of symptomatic fibroids; however, MRI has gained popularity since it is better at characterizing the site and type of fibroid(s) as well as diagnosing other pelvic pathologies such as focal adenomyosis, especially in multi-fibroid uterus or when there is concomitant existence of endometriosis/adenomyosis^23^. MRI allows exact preprocedural planning, whether by myomectomy or by non-invasive treatment such as uterine artery embolization or MRI-guided focused ultrasound^24^.

In summary, accurate advanced imaging is essential in preoperative planning in DIE and other pelvic conditions such as fibroid uteri and adenomyosis in order to have an optimal surgical plan, be able to organize a multidisciplinary team if necessary, and estimate operating time.

In the last decade, the technological advances in ultrasound and MRI machines and the introduction of systematic approaches for the sonographic evaluation of women with suspected endometriosis, such as the IDEA and other protocols mentioned above, have improved the way we use imaging preoperatively. As technology of ultrasound and MRI continues to rapidly evolve, the diagnosis of adenomyosis endometriosis and related pelvic pathologies by these imaging techniques will likely continue to improve.

## Laparoscopic entry techniques

Major complications from gynecological laparoscopy are relatively rare, occurring in three to six per 1,000 cases. Complications related to laparoscopic access represent one third to one half of these adverse events^25^. In about 0.4 of 1,000 laparoscopic procedures, these complications include serious and potentially life-threatening adverse events, such as perforation of the bowel, major abdominal vessels, and vessels of the anterior abdominal wall. These factors make the access phase the most critical step of a laparoscopic procedure. It is important to mention that these numbers have remained largely unchanged over the last two decades despite the introduction of new entry devices and techniques^26^.

There are multiple methods to perform the pneumoperitoneum and insert the trocar into the abdominal cavity: Veress needle insertion for insufflation followed by port placement (a common entry technique used by gynecologists^27^), open laparoscopy (known as the Hasson technique), and direct trocar insertion without previous pneumoperitoneum are some of the most widespread among laparoscopic surgeons. Each of these methods of entry enjoys a certain degree of popularity according to the surgeon’s training, experience, and bias. However, over the years, multiple studies suggest that no one technique shows statistically significant superiority in terms of safety^28^.

A recent Cochrane review^29^ showed that evidence was insufficient to support the use of one laparoscopic entry technique over another. However, it is important to mention that the researchers noted an advantage of direct trocar entry over Veress needle entry for failed entry. Most evidence was of very low quality; the limitations of the study were imprecision (due to small sample sizes and very low event rates) and risk of bias associated with poor reporting of study methods.

In another recent prospective cohort study, Pantoja Garrido *et al*.^30^ compared complication rates and other parameters in direct trocar insertion without previous pneumoperitoneum versus insertion after insufflation with Veress needle. They concluded that direct trocar entry is at least as safe as Veress in regard to the risk of complications arising from access into the abdominal cavity. Compared with Veress, direct trocar entry was found to have some advantages such as a shorter duration of access maneuvers or the lesser number of unsuccessful entry or insufflation attempts.

In summary, although no major changes in regard to entry instruments and techniques occurred in the last decade, we decided to add this section in our review since we believe that the data from recent studies and meta-analyses add to our knowledge and daily practice of laparoscopic entry techniques.

Every entry technique carries some risk of major injury and must be performed with care. Guidelines across the world state that the best technique is the one the surgeon is most familiar with, since they will better understand its associated possible complications and how to identify them as well as ways in which risk can be reduced. Laparoscopy should be performed only when the method of entry is fully understood and when an alternative entry site, method, or both has been identified should they be needed.

Our impression (from speaking to colleagues in Australia and internationally) is that owing to the recent data showing no difference between entry, the direct entry approach seems to be gaining more popularity. It will be interesting to have an updated survey regarding the preferred method of entry by laparoscopic surgeons in gynecology.

## Morcellation in laparoscopic surgery

There has been a shift in the standard of care from a liberal use of open or uncontained morcellation to more restricted use following the US Food and Drug Administration (FDA) review of the topic in 2014. Traditionally, morcellation, whether via mini-laparotomy, vaginal, or laparoscopic, with or without the use of power morcellation, has taken place in an uncontained operative field. However, morcellation has side effects such as spreading occult sarcoma, parasitic myomas^31^, and inherent surgical complications, including potential damage to organs such as bowel or major blood vessels.

In 2014, the FDA stated that laparoscopic power morcellators should not be used to remove fibroid tissue in most women who undergo hysterectomy or myomectomy^32^, causing a significant shift in the clinical practice. As a result, many institutions and health-care systems banned the use of electronic power morcellators in favor of mini-laparotomy and other techniques to take out specimens en bloc^33^.

Results from a survey of gynecological surgeons reported that 61% of providers immediately stopped using power morcellators after the FDA announcement in 2014^34^.

Contained tissue extraction techniques were developed and modified in an effort to minimize the risk of tissue dissemination while allowing for a minimally invasive mode of access and accompanying morbidity advantages^35^.

In February 2020, the FDA updated its safety communication on laparoscopic morcellators, stating that “the FDA recommends performing laparoscopic power morcellation for myomectomy or hysterectomy only with a tissue containment system, legally marketed in the U.S. for use during laparoscopic power morcellation and performing these procedures only in appropriately selected patients”^36^.

Alternatives to uncontained electromechanical morcellation are extracorporeal in-bag manual morcellation through the abdomen or vagina and contained power morcellation. These methods are becoming the standard of care in laparoscopic surgeries, where morcellation is needed^37^.

When comparing vaginal or abdominal manual morcellation with power morcellation, studies demonstrate no differences in perioperative complications^38–40^. Cohen *et al*.^41^ prospectively compared contained manual morcellation techniques and found no differences in blood loss, operating time, length of stay, or complications between abdominal mini-laparotomy and vaginal tissue extraction but did find a significant difference in bag leakage (8.3% in mini-laparotomy versus 40.6% in vaginal tissue extraction), which is consistent with the published literature^42^. The clinical significance of bag disruption during tissue extraction is unknown.

A recent Cochrane systematic review^43^ looked at the effectiveness and safety of in-bag manual morcellation compared with uncontained power morcellation during laparoscopic myomectomy. Two randomized controlled trials (RCTs), which enrolled 176 premenopausal women undergoing laparoscopic myomectomy, were included and neither study reported complications, such as diagnoses of leiomyosarcoma, during or after surgery for women in either group. The authors concluded that they are uncertain whether in-bag morcellation reduces the total time of the operation or improves the ease of morcellation. They also mentioned that the quality of the evidence was extremely low.

Six years after the initial FDA warning, it seems that the gynecological surgical community is still adapting to the new practices through innovation and research. The rates of laparoscopic procedures using morcellation have recovered and continue to rise^44^. Surgeons and manufacturers have created tissue extraction containment systems to replace the electromechanical morcellator to limit tissue dissemination but permit a minimally invasive surgical approach. The containment systems are widely used by surgeons; however, it is important to mention that only a few are FDA-approved for this indication. Ongoing research to understand the safety of these containment systems is necessary to avoid future controversy.

## Barbed sutures in laparoscopic surgery

In recent years, a new class of suture material—the barbed suture—has been introduced into the surgeon’s armamentarium and is gaining popularity, especially in laparoscopic and robot-assisted surgery, particularly in cases requiring extensive suturing. Modern barbed suture can trace its origins to John H. Alcamo, whose idea was issued a US patent in 1964 for “a suture so formed that it prevents slippage in sutured incisions or wounds”^45^. Despite some early usage of barbed sutures and evolution of early designs over the years, it was not widely used or manufactured until 2004, when the Quill Knotless Tissue Closure Device (Angiotech Pharmaceuticals, Vancouver, BC, Canada) was approved by the FDA^46^.

Nowadays, the three commercially available barbed sutures are the V-Loc Absorbable Wound Closure Device (Covidien Healthcare, Mansfield, MA, USA), the Quill Self-Retaining System (Angiotech Pharmaceuticals), and the Stratafix (Ethicon Endosurgery, Somerville, NJ, USA)^47^.

Barbed sutures come in many forms and shapes. They can have a needle at one end (unidirectional barbed suture) or needles at both ends with a change in barb direction in the midline of the suture (bidirectional barbed suture). They are usually made of an absorbable monofilament, but non-absorbable sutures are available in the market as well.

The barbed sutures facilitate the operative procedure by maintaining the tensile strength of the suture and by providing an even distribution of tension along the closure^48^. Tension is distributed not just at the knots but along the entire length of the suture^49^.

The introduction of knotless barbed sutures into the surgical market has decreased the challenges of laparoscopic suturing. Since their introduction in 2008 by Greenberg and Einarsson^50^, barbed sutures have become more popular among minimally invasive gynecologic surgeons and are frequently the suture of choice for closing the vaginal cuff during total laparoscopic hysterectomy, re-approximating the myometrium after laparoscopic myomectomy, and reducing the procedure time of laparoscopic sacrocolpopexy^51^.

### Barbed sutures in laparoscopic myomectomy

One of the most common procedures performed by gynecologists using barbed sutures is laparoscopic myomectomy. Suturing uterine wall defects is considered one of the most important factors influencing the surgical outcome in a myomectomy: it is essential to avoid oozing or hemorrhage, the formation of dead space in the myometrium, and the subsequent formation of uterine hematomas that might increase the complication rate^52^. To this end, barbed suture materials are potentially the ideal solution. Their synthetic, monofilament configurations should minimize local inflammation, and the operation time is reduced significantly when using them^46^.

Historically, uterine muscular wall defects were sutured by absorbable filaments (continuous or interrupted). Obviously, great surgical abilities and experience are required to perform intracorporeal knots during laparoscopic myomectomy while maintaining suture tension during the closure of the myometrium.

A recent meta-analysis by Gardella *et al*.^53^ showed that compared with conventional sutures, barbed sutures demonstrated significant reductions in suturing time, operating time, intraoperative blood loss, postoperative hemoglobin drop, length of hospitalization, and even complication rates. These results reinforced the conclusion of a previous meta-analysis that showed similar results^54^.

In regard to the effect of barbed sutures on fertility and pregnancy after myomectomy, a recent retrospective cohort study and follow-up survey by Pepin *et al*.^55^ showed that pregnancy outcomes after laparoscopic myomectomy with barbed suture are comparable to the available literature on pregnancy outcomes with conventional smooth sutures, making the former safe to use in procedures that are indicated to improve fertility and obstetrical outcomes.

### Barbed sutures in laparoscopic hysterectomy

In the altered environments of laparoscopic and robotic hysterectomies, cuff closure with barbed sutures has flourished and has become the standard of care in many institutions. The reduced operative times and simplicity of the closure make the use of barbed suture a good choice for this application.

In recent years, several studies have shown that vaginal cuff closure performed with barbed suture material is a safe and well-tolerated procedure and reduces operative times with reduced or no change in the incidence of vaginal cuff dehiscence compared with monofilament non-barbed suture^56–60^. In combination, these studies confirm that the use of barbed sutures for vault closure in laparoscopic hysterectomy is both convenient and safe.

### Other applications for barbed sutures in laparoscopic surgeries

Other laparoscopic procedures in which barbed sutures were described are laparoscopic/robotic sacrocolpopexy, SILSs (also known as laparoendoscopic single-site surgery, or LESS) hysterectomy/myomectomy, and laparoscopic uterosacral suspension^61–64^. It is important to mention an infrequent but potential harmful complication seen with the use of barbed sutures: small bowel obstruction. Several case reports had previously reported the occurrence of small bowel obstructions after laparoscopic myomectomies, hysterectomies, and sacrocolpopexies^65–67^. These complications are a result of the remnant length of the barbed suture entrapping the small bowel and can be minimized by making sure that any exposed end of the suture is as short as possible. Some experts have suggested cutting the suture flush with the peritoneum, which is what the product’s package inserts and directions for use also recommend^68,69^.

In conclusion, there is no doubt that the incorporation of barbed sutures into advanced laparoscopic surgeries, mainly laparoscopic myomectomy and laparoscopic hysterectomy, revolutionized the way these procedures are being performed. The clinical literature provides evidence that the performance of absorbable barbed sutures is at least equivalent to that of conventional absorbable smooth sutures for soft tissue approximation. In addition, the literature suggests that the use of barbed sutures can shorten surgical time and possibly reduce intraoperative blood loss. With the introduction of newer barbed suture products, the applications of this evolving technology will undoubtedly expand, although additional randomized clinical trials are needed to better elucidate its full potential.

## Emerging minimally invasive techniques: single-incision laparoscopic surgery and transvaginal natural orifice transluminal endoscopic surgery

Laparoscopic surgery has been widely adopted in gynecology because of numerous benefits compared with open surgery^70^. The quest for approaches that are even less invasive has led to the advent of techniques such as the SILS (also called LESS) or the more recently introduced vNOTES.

### Single-incision laparoscopic surgery 

The use of single-incision laparoscopy in gynecology was first described in the 1970s, when it was used to perform tubal ligation in an outpatient setting^71^. Two decades later, Pelosi and Pelosi^72^ reported the first single-incision subtotal laparoscopic hysterectomy.

Single-port laparoscopic surgery is generally considered to be more difficult to perform than conventional laparoscopic surgery. The most important factor is the limited number of simultaneously available surgical instruments and collisions between the instruments because of the lack of triangulation caused by their proximity to each other^73^.

Recent advances in technological innovation (multi-channel single-port systems, articulating instruments, and high-definition visualization) have made most laparoscopic procedures feasible by the SILS approach. Currently, hysterectomy, myomectomy, and adnexal surgeries are well described and are performed by various surgeons worldwide. However, questions remain about comparative effectiveness with respect to complications, operative time, blood loss, postoperative pain, length of hospital stay, and cosmesis^73^.

In 2017, Schmitt *et al*. reported a meta-analysis evaluating the clinical advantages of SILS for adnexal surgery^74^. Through a pooled analysis of six RCTs, the authors showed that there was no significant difference between SILS and conventional multiport laparoscopy regarding duration of hospital stay, blood loss, postoperative pain, and cosmetic outcomes. In fact, SILS was associated with a longer operative time. Because there were no significant differences found between SILS and conventional multiport laparoscopic surgery, the authors concluded that SILS cannot be recommended for adnexal surgery.

Owing to the specific challenges related to myomectomy, SILS myomectomy was introduced relatively recently. Myomectomy requires more suturing and knot-tying than do other benign operations. Also, securing sufficient traction in the process of separating myomas from the uterine bed is a laborious procedure^75^.

In a recent meta-analysis^76^, two RCTs and six observational studies were analyzed to compare the surgical outcomes of SILS myomectomy with those of conventional multiport laparoscopy. SILS myomectomy was comparable to conventional multiport laparoscopy in terms of safety and feasibility when patients were selected according to the inclusion criteria regarding the size and number of the myomas. SILS myomectomy was found to be more advantageous in terms of immediate postoperative pain. Moreover, myomas may be removed more quickly and safely from the abdominal cavity in SILS than in conventional multiport laparoscopy because of the larger umbilical port. A dedicated umbilical port such as the GelPOINT Mini Advanced Access Platform (Applied Medical, Rancho Santa Margarita, CA, USA) can be used with a contained extraction system such as the Alexis Contained Extraction System (Applied Medical) to perform contained manual morcellation.

SILS hysterectomy is considered a challenging procedure, especially due to the difficulty of vault suturing, compared with conventional multiport laparoscopic hysterectomy. Many studies of SILS hysterectomies use barbed sutures for vaginal cuff closure because these materials eliminate the need for intracorporeal knot-tying, which is particularly demanding in SILS^73^.

Shin *et al*.^77^ described the closure of the vaginal cuff during single-site hysterectomy using a V-Loc unidirectional barbed suture with a straightened needle. They found that the use of a straightened needle shortened operative time and reduced technical difficulty during this procedure^77^.

### Transvaginal natural orifice transluminal endoscopy surgery

NOTES is a scarless single-entry procedure, a novel technique in minimally invasive surgery. There are a number of different access routes for NOTES, but the vagina has been under particular scrutiny because it may be the safest and most feasibly applied^78^.

The journey to NOTES began long before the endoscope was invented. Vaginal hysterectomy has long been regarded as the original natural orifice transluminal surgery; a reported attempt has been traced back to Soranus of Ephesus (120 CE)^79^.

Potential advantages of NOTES include the absence of a visible abdominal scar, less operative pain, shorter hospital stay, improved operative visibility, and no requirement of adhesiolysis to expose the pelvic organs^80^.

The current enthusiasm for vNOTES is rapidly growing, and thus far the concept has shown exciting potential as vNOTES adnexectomy, hysterectomy, and sacrocolpopexy were described and are carried out in selected sites worldwide.

Recently, a meta-analysis evaluated the advantages and disadvantages of vNOTES hysterectomy in patients with benign gynecological disease^81^. The study did not find RCTs but included two retrospective cohort studies comparing vNOTES hysterectomy with conventional laparoscopic assisted vaginal hysterectomy (LAVH) (either SILS or multiport laparoscopy LAVH). Compared with conventional LAVH, the vNOTES group was associated with shorter operative time and hospital stay but higher cost. There were no differences between the groups in terms of intra- or postoperative complications and postoperative pain.

In summary, SILS and vNOTES are both emerging techniques in the evolution of minimally invasive surgery. Their technical limitations continue to lessen with the gain of experience and recent technological innovations, such as various types of multi-channel ports, better optical instruments, and articulating instruments.

The feasibility and safety of SILS for the treatment of many of the benign gynecological diseases have been demonstrated. However, the evidence is not strong enough to recommend the use of SILS over conventional multiport laparoscopy, and the decision of whether to perform a procedure by SILS or by conventional multiport laparoscopy should be based on surgeon experience, proper patient selection, and patient preference.

And although vNOTES represents a significant innovation in gynecological surgery, there are still technical limitations that must be overcome before widespread use of this approach can be considered. Since much of the current data come from very few centers, there is a need for more studies to be carried out in various centers with various surgeons in order to have better knowledge about the feasibility and safety of this novel approach.

## Enhanced recovery after surgery in minimally invasive gynecological surgery

Surgical postoperative management typically consisted of a “wait and see” approach, whereby the surgeon responds to the patient’s needs postoperatively. Many traditional aspects of perioperative care have insufficient data to support them. In 2003, Kehlet and Dahl^82^ proposed an active attitude, which is now known as enhanced recovery after surgery (ERAS), that has led to a paradigm shift in clinical practice.

ERAS is a bundled pathway based on evidence-based practices with the goal of hastening recovery^83^. ERAS protocols include preoperative, intraoperative, and postoperative interventions and have been shown in multiple investigations to accelerate return of bowel function, reduce opioid use, decrease hospital length of stay, and reduce costs with high patient satisfaction after gynecological surgery^84,85^. Most of the literature investigating ERAS in gynecology has focused on open gynecological surgery; recently, however, growing evidence shows the advantages of ERAS in minimally invasive surgery as well.

In 2006, a formalized, evidence-based guideline for patients undergoing gynecological surgery was published by the ERAS Society^86,87^; in 2019, an update was released^88^.

A systematic review by Kalogera *et al*.^89^ identified 12 studies reporting outcomes specific to ERAS in minimally invasive surgery and one study on ERAS in minimally invasive surgery combined with bowel surgery. It is important to mention that all studies assessed ERAS around laparoscopic hysterectomy. In keeping with findings for ERAS in open gynecological surgery, ERAS pathways shortened hospital length of stay and/or increased the proportion of same-day discharges, improved patient satisfaction, and significantly reduced hospital costs without increasing postoperative complications or readmission rates.

In a retrospective study that looked at outcomes in minimally invasive non-hysterectomy gynecological procedures, Peters *et al*.^90^ showed that implementation of ERAS resulted in increased same-day discharge rates and improved perioperative outcomes, including reduction in opioid usage, without affecting 30-day morbidity in women undergoing laparoscopic minimally invasive non-hysterectomy gynecological procedures. The authors concluded that ERAS should be considered for all gynecological laparoscopic procedures, not only hysterectomies.

In a recent randomized trial, Yilmaz *et al*.^91^ aimed to investigate the impact of ERAS in patients undergoing minor gynecological surgical procedures (laparoscopic and hysteroscopic). One hundred and four patients were randomly allocated to one of the following study groups: the ERAS group or the conventional care group, which consisted of age-matched control patients who did not receive ERAS interventions. The implementation of the ERAS protocol, compared with conventional care, led to significantly shorter length of stay, early mobilization, and reduced fluid intake without an increase in complication rate.

ERAS has been shown to significantly lower perioperative costs as well. Early cost analysis has shown significant cost savings after ERAS implementation for specific operations, such as colorectal procedures, cystectomy, pancreatectomy, or hepatectomy^92,93^.

As for the role of ERAS in cost reduction in gynecological surgeries, a recent retrospective Swiss study by Pache *et al*.^94^ compared perioperative costs between consecutive patient groups undergoing gynecological surgery prior to, immediately after, and three years after ERAS implementation at a single center. The conclusion was that implementation of ERAS in gynecological surgery induced a significant and sustained decrease of overall costs during the first three years after implementation.

In conclusion, since the introduction of the concept of fast-track surgery and the subsequent development of ERAS pathways in multiple specialties, a growing body of high-quality literature has continued to support the adoption of ERAS in all subspecialties of gynecological surgery, including minimally invasive surgeries, and implementation of this revolutionary approach to modern surgical management is expected to increase over the next several years.

## Role of simulation in laparoscopic surgery training 

The surgical skill set to carry out endoscopic surgery is essentially different from that of open surgery, creating a steeper learning curve. The specialized equipment and instrumentation require a different set of technical skills: significant hand–eye coordination and optimal psychomotor skills are essential requirements to be a skilled laparoscopic surgeon. Lack of 3D visualization, loss of tactile feedback, and counterintuitive movement of instruments caused by the fulcrum effect are just some of the obstacles for trainees to master the art^95^. Moreover, the opportunities for trainees to master these techniques in the traditional apprenticeship model have diminished. This is due partly to reductions in trainees’ working hours and partly to the increased use of conservative management for common gynecological problems (for example, methotrexate for ectopic pregnancy, the progesterone-secreting intrauterine systems, and ablation techniques for heavy menstrual bleeding), which has resulted in fewer surgical interventions and thus training opportunities^96^.

Together with the exponential growth of technology and approaches, the traditional model of gynecological surgical training had to rapidly adapt, compared with what it was up until only two decades ago. The “see one, do one, teach one” approach to assimilating surgical skills is no longer an accepted approach. Hence, new solutions for training, such as structured curricula, including theory, simulation, and the development of dedicated fellowship programs for advanced training in minimally invasive surgery, are evolving^97–99^.

One of the biggest advances in training in laparoscopic surgery in the past decade is the incorporation of simulation to medical training and credentialing.

Simulation-based medical education has been identified as a potential solution to address deficits in laparoscopic surgical training. Fortunately, with current technological advances, almost all modalities of laparoscopic surgery are amenable to improvements in surgical education via simulation^100^. Surgical simulation also offers a platform for objective assessment of selected surgical skills. Studies have clearly shown that simulation-based medical education with deliberate practice is superior to traditional clinical medical education in achieving specific clinical skill acquisition goals^101^.

### Low- and high-fidelity simulators 

The growing integration of low-fidelity (basic simulations such as box trainers) and high-fidelity (advanced simulation by virtual reality techniques or live tissue) simulation training in laparoscopic surgery has led to improved skill acquisition^102–104^. A well-established low-fidelity simulation model is the fundamentals-of-laparoscopic-surgery module, through which trainees are taught vital psychomotor skills via a validated box trainer that is supported by a cognitive component^105,106^.

The advent of laparoscopic virtual reality training systems has raised the learning potential further, even for experienced surgeons. Some potential benefits of virtual reality simulation in laparoscopic surgery include education on an interactive 3D pelvis, step-by-step procedural guidance, a comprehensive return of performance metrics on vital laparoscopic skills, and the incorporation of advanced skills such as laparoscopic suturing, complex dissections, and lysis of adhesions.

Generally, high-fidelity computerized simulators provide a comprehensive performance report on completion of training, along with a complete recording of the trainee’s encounter during skill acquisition. Most importantly, laparoscopic training via simulation has been validated to translate into improved operating room performance by impacting operating times, safety profiles, and surgical skill growth^97,107^.

### Other simulation modalities

***Organic simulators***. Organic simulators are termed “high fidelity” as they approach real-life situations. Human cadavers are ideal in terms of anatomy and tissue consistency; however, tissue fidelity is lower than in live models, it is not possible to simulate complications such as bleeding, they cannot be used more than once, there is the potential for disease transmission and ethical concerns, which means that cadavers are of limited use, especially for the teaching of anatomy and anatomy-based surgical approaches.

***Animal models***. Animal models provide realism during operative training and provide good practice in the maintenance of hemostasis and mimic complications, but they have anatomical differences from the human body. The uteruses, fallopian tubes, and ovaries of other mammals only minimally resemble those of women, which substantially limits organic animal-based simulation of minimally invasive procedures.

***Hybrid trainers.*** Hybrid trainers combine virtual reality with video box simulation, provide realistic haptic feedback as in actual surgery, and give metrics without the need for the presence of an experienced surgeon to give feedback to the trainee. Real instruments are inserted through specific holes and enable manipulation of physical objects in a box simulator. Examples of hybrid trainers are the ProMIS (Haptica Inc., Dublin, Ireland), which aims at the training of basic minimally invasive surgical skills, including suturing and knot-tying, and the LapTrainer with SimuVision (Simulab Inc., Seattle, WA, USA). This is an open box trainer using a simulated laparoscope (SimuVision) and has four standardized exercises ranging from basic to more advanced^108^.**

***Augmented reality laparoscopic simulator.*** Augmented reality (AR) refers to a system that overlays computer graphics images and real video images into a single perception of an enhanced world around the user, connecting both the virtual and the real world. In AR laparoscopy, the laparoscopic task is demonstrated on a screen, and the trainee is objectively assessed post-performance without an expert laparoscopic surgeon needing to observe and guide the trainee. In the last couple of decades, several AR simulators, such as the ProMIS AR laparoscopic simulator, have been developed^109,110^.

No tool or method is clearly superior over another for skill acquisition, even though a variety of simulation tools as well as methods for training and testing specific endoscopic skills are available. In a systematic review, Torres-de la Roche *et al*.^98^ showed that well-guided training courses combined with different trainers and methods significantly improve a surgeon’s laparoscopic skills and suturing ability, which are unforgettable over time. However, this proficiency could deteriorate over time when it is learned and executed solely on simulation trainers. The authors concluded by stating that structured curricula, including theory, simulation, and live surgery, seem to be the best options for trainees^98^.

### Maintenance and credentialing of laparoscopic skills

Equally important to basic skills simulation training is the process by which a trained individual can obtain the appropriate credentials and subsequent privileging to perform various surgical procedures. Simulation has begun to play a significant role not only in an individual’s initial credentialing and privileging in surgery but also in maintaining those privileges.

Using simulation for recertification has been criticized because, although it can confirm that a surgeon is skilled enough to operate the tool, it does not evaluate surgical judgment or technique. One potential solution to overcome this issue is to have, in addition to simulations, a crowdsourced review of an individual surgeon’s surgical videos. This has proven to be a useful and dependable way to give a surgeon direct feedback regarding his or her performance on a live patient^111^.

Some institutions use this technology for initial training as well as to help surgeons improve by providing direct feedback from expert surgeon reviewers. Others have considered using this technology in place of annual re-credentialing case volume requirements, which may enable a more accurate assessment of competence^112^.

In summary, owing to advances in simulation technologies and the revolution in surgical education, the training and annual recertification of future surgeons have changed significantly over the past decade. We expect that, together with structured curricula and the development of dedicated fellowship programs for advance training in minimally invasive surgery, simulation will further develop and become even more dominant in training and annual recertification, similar to the simulation training and recertification that aircraft pilots are required to follow.

## Challenges and limitations in laparoscopic surgery

A thorough discussion of the technological advances and the widespread use of laparoscopic surgery will never be complete without mentioning at least some of the main challenges and limitations that affect this rapidly evolving field. The benefits of minimally invasive surgery over traditional laparotomy have been demonstrated in a multitude of studies, as discussed earlier, and in the hands of trained laparoscopic surgeons, minimally invasive approaches are considered equal or superior to laparotomy for the treatment of most benign gynecological diseases^113–115^.

However, despite a general steady increase in the use of minimally invasive techniques, there are still many barriers for the uptake of minimally invasive surgery in certain settings.

It has been reported that, in the US, fewer than 60% of benign ovarian masses in adolescents^116^ are treated by laparoscopy and a large number of hysterectomies for benign conditions are still being performed by open surgery^117^ and that, in the UK, the rate of total laparoscopic hysterectomy was approximately 23.6% of the total number performed in 2015–2016^118^.

Some of the main barriers to the adoption of laparoscopic surgery over an open approach are surgeon preferences and training. Surgeon preferences are formed through experience and interpretation of the evidence base, and they vary widely among clinicians. Core medical training is an important influence in the development of a clinician’s preferred techniques, as well as when and from whom they received their training (more recently trained practitioners are usually more familiar with and open to laparoscopic surgery).

In a survey on barriers to the adoption of laparoscopic surgery, Fuchs Weizman *et al*. highlighted several key challenges that are potential limitations to the use of laparoscopy in benign gynecological surgery: lack of case volume (especially relevant to small and rural hospitals where surgical volumes are low), discomfort with unexpected scenarios, video–eye–hand coordination, depth perception, and laparoscopic suturing^119^.

Training and a lack of suitably trained clinical staff are key factors identified in the literature^118^ as barriers to the uptake of laparoscopic surgery. Although sufficient provision of training courses for minimally invasive surgery seems to be almost universal in most developed countries, there is divergence relating to how clinicians can access this training. Among the issues are funding and access to funding, and some clinicians fund their own training. Another factor is the ability for surgeons to practice their skills in settings where patient throughput is insufficient^119^.

This brings us to the growing understanding that the treatment of complex benign gynecological conditions requires advanced laparoscopic skills, which are best served by specialized advanced training. Several training programs worldwide offer a dedicated advanced minimally invasive surgery fellowship^120–122^, and some societies even stipulate that the laparoscopic treatment of complex conditions can be provided only by laparoscopic surgeons who completed an accredited advanced laparoscopic fellowship or other form of advance training^123^.

### Silo thinking in the management of resources

As discussed by Cole *et al*.^118^, “invest to save”—specifically investing in the theatre time and equipment to deliver minimally invasive surgery—is outweighed by the savings generated by improving recovery and shortening length of stay. However, the costs/benefits may be drawn/accrued from different budgets, and so there is a necessary oversight needed to balance the costs and savings associated with the introduction of minimally invasive surgery. In turn, this system-wide approach must translate to individual decision-making. Evidence indicates that the oversight needed to overcome silo budgeting may collapse when attaining short-term targets and contradictory objectives are the primary influences in making everyday decisions.

A recent example for the cost-effectiveness of laparoscopic surgery is a study by Capozzi *et al*., who compared laparoscopy versus laparotomy for hysterectomy in obese women^124^. The investigators suggested that minimally invasive surgery is more advantageous in terms of both costs and postoperative complications and concluded that laparoscopic surgery in obese patients allows an economic saving of about 60% (compared with open surgery), mainly by reducing pre- and postoperative tests and evaluations and by reducing the length of inpatient stay postoperatively.

### Laparoscopy in developing countries 

Even though laparoscopy has thrived in high-income countries, it is still inaccessible for the majority of people around the world, who live in low- and middle-income countries (LMICs). Laparoscopy has the potential to have a greater benefit in terms of mortality and morbidity in areas such as LMICs, where there may not be access to clean water, sanitation, blood banks, advanced diagnostic imaging, or interventional radiological procedural services^125,126^.

Laparoscopy programs in LMICs must overcome various obstacles, such as an inadequate number of skilled providers; longer operating time; insufficient resources, equipment, and maintenance capacity; and no safe procedure guidelines^127,128^. Although it is widely thought that a lack of resources and training is the reason, recent evidence found that there may be other barriers. Choy *et al*.^129^ showed that other barriers hindering the adoption of laparoscopy in developing countries are (1) the organizational structure for funding laparoscopic procedures (ongoing funding structure, rather than upfront costs, may limit the number of cases done), (2) the hierarchical nature of the local surgical culture in these countries, and (3) the expertise and skills associated with a change in practice. (Owing to the generalist nature of surgical practice, surgeons are less willing to practice more technically complicated and time-consuming procedures).

There is no doubt that, as the world’s population is growing constantly (and at a greater rate in developing countries), overcoming the barriers to the uptake of laparoscopic surgery is becoming a big challenge. Rigorous programmatic evaluation; involvement of key community, government, and health-care stakeholders; and development of stable financing options are just some of the required solutions when technology is moved across socioeconomic, cultural, and geographic boundaries.

## Conclusions

During the last two decades, minimally invasive surgical techniques have revolutionized the field of gynecological surgery. A myriad of evidence has demonstrated the improvement in patient outcomes associated with a minimally invasive approach compared with open surgery.

In this article, we have reviewed the current literature on some minimally invasive surgical innovations, advances, and common practices in benign gynecology that made an impact on the way laparoscopic surgery is performed and managed in the last decade.

Some of the advances in laparoscopic surgical management and outcomes are the result of the rapid evolution in perioperative management. Improvements in preoperative imaging and the incorporation of ERAS to laparoscopic surgery in gynecology have had a significant role in improving efficiency, safety, and patient satisfaction.

More evidence on laparoscopic entry techniques from recent years has shown that there is no one entry technique superior to the others and that the three common entry techniques—direct entry, Veress entry, and open (Hasson)—are equally safe and effective.

Barbed sutures have revolutionized the way we perform intra-abdominal suturing and have made operations such as hysterectomies and myomectomies safer, more feasible, and more efficient compared with just a decade ago.

The approach to tissue morcellation has changed dramatically in the past six years. Now, after the introduction of new contained morcellation instruments and techniques, the rates of laparoscopic procedures using morcellation have recovered and continue to rise. Ongoing research to understand the safety of these containment systems is necessary to make contained morcellation safer, easier, and faster.

Although their value over conventional laparoscopy remains unproven, SILS and vNOTES provide an opportunity to perform complex surgeries through approaches that are even less invasive than traditional techniques. As evidence for use of these novel technologies continues to mature, the utility of these approaches as well as the optimal patient and case selection will need further evaluation.

As simulation technologies matured in the last decade, the training and annual recertification of future surgeons have changed significantly and now closely mimic the pathway that all airplane pilots are required to follow:

- *Initial training* will require mastery of surgical techniques using a simulator before taking a “solo flight” on a live patient.
- *Maintenance of privileges* now requires either large case volumes or skills testing on a simulator. Many institutions now also require an annual “check ride”, such as a crowdsourced video review of a surgeon’s cases.
- *Re-credentialing*: An annual objective evaluation of good surgical judgment and surgical technique proficiency will certainly be required in the future.

Despite the benefits of minimally invasive surgery over traditional laparotomy, there are still some major barriers for the uptake of minimally invasive surgery both in Western countries and in developing countries, and overcoming these barriers is a growing challenge.

We should remember that improvements in operative outcomes for the patient should ultimately be of utmost importance and that as novel surgical modalities are rapidly introduced, objective evidence must be constantly sought and published to support assimilation into the field without doing any harm—*Primum non nocere!*
